# Progress in Medicine

**Published:** 1897-12-25

**Authors:** 


### PROGRESS IN MEDICINE.

### FEVERS.

(*Continued from page* 204.)

**Complications of Typhoid.**—Jaccoud[1] describes what he calls "sweating typhoid," as common in southern Italy and France. It is not, however, clear why it is called typhoid; no case has ever been fatal, or produced perforation. The rose spots may or may not be seen, and the fever lasts five weeks with daily rigors and sweats. The temperature may be intermittent for the first week, and throughout shows a daily rise before the sweating. It is quite distinct from malaria and influenza. The difficulty of deciding what are the most reliable tests for typhoid is shown by a case, without any ulceration of the bowels, recorded by Dr. Cheadle.[2] The patient, a boy of three years, had a brother suffering from typhoid, and died on the thirty-second day from heart failure. Not only was the Widal reaction positive, but bacilli were isolated from the fæces and urine. No ulceration, however, was found, and the Peyer's patches were normal, but the mesenteric glands were enlarged, and the clinical symptoms were of the ordinary typhoid type. Remlinger[3] met with an instance of liver abscess and empyæma complicating an ordinary typhoid, and succeeded in finding the bacillus in both fluids. Those from the liver abscess had but little virulence, and indeed that organ seems to have the power of attenuating any microbe inhabiting it. The author thinks it strange that abscesses of the liver so rarely accompany typhoid, considering the frequency of ulceration of the large intestine. In this case the colon was normal, but the abscess contained the typhoid bacillus which also existed in the intestines, and especially in Peyer's patches. It has also been found as a pure culture in the pus of an inflamed wrist joint, during the third week of the disease, by Martin and Robinson.[4] In most suppurations during enteric the ordinary pyogenetic organisms are present, but here they were entirely absent, so that it is clear that Eberth's bacilli have the power of producing pus even when alone. It has been asked whether in their wanderings over the body they can attack the fœtus in utero. Tubercle, variola, and possibly anthrax can be thus communicated. Crozier Griffiths[5] endeavours to prove this from the fact that a child born during its mother's illness from typhoid gave the Widal reaction seven weeks after birth; but it is pointed out that the mother's milk might convey to it the infective material, or, what is still more likely, the placental blood was charged with microbicidal material, if not with true anti-toxin. A number of persons have recently been inoculated with Wright's protective fluid, and then gave the Widal reaction readily enough. Bermano[6] investigated another form of infection, that from dust carried through the air. Some evidence has been thought to show that the bacilli have been transmitted as much as a mile in this way, but the author proved that if once dried the microbes were killed. Still he kept them alive for many days, as long as the earth or fæces remained damp. Indeed, pieces of cloth, moistened with typhoid bouillon and kept in a dwelling-room, showed living germs after two months. Thus, articles soiled by typhoid patients may retain infective powers for unlimited periods if kept in a half-dry state. For disinfecting the stools, Gilman Thompson[7] advises a solution of one in ten crude carbolic, which should be thoroughly mixed with the stool, it being left in contact for two hours or more. The bed linen should be soaked in a one in twenty solution, and afterwards boiled. The patient's perineum should be sponged with a sublimate solution, and the nurse should wash her hands, as well as the thermometer, enema syringe, and other utensils, with a similar strong solution. Where no drainage system exists, chloride of lime should be mixed with the excreta.

**Treatment.**—Weispecker[8] reports twelve cases of typhoid, scarlatina, and pneumonia treated by serum from convalescents. The two typhoids showed a rapid fall of temperature and general improvement. Sailer, on the other hand, observed no results at all when similar trials were made at the Philadelphia Hospital with great care and large quantities of serum. H. Duchenne[9] administers to his patients large quantities of bland liquids by the mouth, and a tepid enema daily. Thus he gives milk and as much of simple flavoured waters as he can get the patient to drink, and reports that, under this treatment, convalescence is usually reached in three weeks, and that he has attained a very low rate of mortality by its use. Mueller[10] treated thirteen cases with perchloride of mercury and an initial dose of calomel, and claims for it a rapid reduction of temperature if commenced at an early stage, and a marked improvement and steady progress in all. If the temperature rose, the perchloride was given more frequently. He fed his patients entirely on broths, jellies, and eggs, without any milk at all, until the temperature remained normal, and no tenderness existed in the abdomen.

Among recent records we notice that Ohlmacher[11] found the bacilli in the meninges in two cases of meningitis in typhoid; while McCormick treated a hundred cases with only one death. He orders a milk and broth diet, a tepid bath twice a day, calomel every two hours until four to eight motions are passed, and then two minims of guiacol every two hours. Water is given freely by the mouth, and if hæmorrhage occurs opium is forbidden. Osler, in reply to his paper, declared opium was valuable in this emergency, and pointed out that, while others like Stewart, in Montreal, had had a series of even 130 cases without a death, McCormick's treatment was followed by a severe percentage of hæmorrhages. Tyson[12] shows the value of bathing by daily estimations of the urea excreted in a case complicated by broncho-pneumonia and mild nephritis. The patient had 53 baths, and the albumen for a time reached a quarter of the bulk of the tube; but the urine increased with the baths, and the excretion of urea with it. The estimation was continued for 30 days, and showed throughout a large part of the time as much as 400 and 500 grains of urea daily. The possible amount due to the milk food given, if all was digested, would amount to 380 grains. All complications disappeared, and the patient made a good recovery. Surgeon-Captain Thacker[13] advocates a modification of Quill's carbolic treatment, giving 12 minims of pure carbolic, divided into four doses, in the 24 hours, in plenty of water, and enemata of 10 grains each of Dover's powder and tannin when hæmorrhage takes place, with acid, opium, and tannin by the mouth. He finds that the carbolic treatment reduces the temperature and the fermentation in the bowels, and cleans the tongue. Gautier,[14] in two almost hopeless cases with cerebral symptoms, formed artificial abscesses by the injection of turpentine into the thigh. The "typhoid state" passed away, the cerebral symptoms slowly vanished, and both patients recovered. Cutler,[15] after reviewing the various systems recommended, falls back on milk diet and morphia freely given to allay nervous excitement, and whisky, well diluted, daily.

**Scarlatina.**—Pettidi[16] has employed inunctions of pilocarpine as a remedy for scarlatinal nephritis in an adult. After dry cupping the loins, an ointment containing half a grain of pilocarpine to the ounce was rubbed in, and the area covered with a thick layer of cotton. In six days of this treatment the urine once more became very abundant and clear, The application was continued for twenty-five days, and the secretion became perfectly normal. Fussell[17] inquires what is the characteristic tongue in scarlatina? and finds a general agreement among German and English and French authorities that for the first few days it is white, coated with prominent papillæ, and that on the third or fourth day this desquamates, leaving the tongue bright red with prominent papillæ. It is its latter stage, he thinks, which is peculiar to scarlatina, and to it alone, he, and many American writers, would give the name of "strawberry," a name which was first employed in 1845, probably by Watson.

[1] B.M.J., Mar. 20. [2] Lancet, July 31. [3] Med. Bulletin, July. [4] Amer. J. Med. Sc., Sept. [5] Lancet, June 26. [6] Med. Press, Sept. 29. [7] Med. Rec., Sept. 4. [8] Inter. Med. Mag., July. [9] Amer. J. Med. Sc., July. [10] Australas. Med. Gaz., July 20. [11] Med. Rec., July 3. [12] Am. J. Med. Sc., Sept. [13] B. Med. Jour.. May 29. [14] B. Med. Jour., June 26. [15] N. Y. Med. Jour., June 12. [16] N. Y. Med. Jour., Ap. 17. [17] N. Y. Med. Jour., May 22.

### DISEASES OF THE LUNGS.

(*Continued from page* 203).

The prognosis of pneumothorax in any given case must be founded in large part on the above facts. There are three points to be considered. First, the immediate risk to life. This is always great, and greatest during the earliest hours and days; in considering it the following points are of importance: (*a*) The urgency of the symptoms, which is measured practically by the amount of dyspnœa and cyanosis. The greater the relief given by paracentesis, and the longer the interval between successive paracenteses, the better the immediate prognosis. (*b*) The condition of the opposite lung; if this gives way under the strain, signs of bronchitis manifest themselves. (*c*) The effect on the heart. If too much work is thrown on the heart dilatation results. (*d*) The general strength of the patient. (*e*) The disease causing the pneumothorax. This is usually phthisis; if it is simple injury the prognosis is better; if it is gangrene of the lung the prognosis is almost always fatal. The prognosis as to the duration of life, supposing the initial symptoms have passed off, depends on the general strength of the patient and on the original disease, but principally on the complications which may ensue, *i.e.*, on the occurrence of effusion and its character. The number of recoveries is small, about 25 per cent. The most favourable are those in which no effusion takes place; if a serous effusion forms recovery takes place with or without paracentesis, whilst if the effusion be purulent in character the prognosis is almost hopeless, though very occasionally even then recovery takes place. That the prognosis even in pyopneumothorax from gangrene is not necessarily fatal is well shown by a case recorded by Ewart and Sheild.[19]

Chaufford[20] gives an account of a case of simple pneumothorax and its diagnosis by tuberculin. The patient, a woman aged 30 years, developed a pneumothorax as a result of a sudden muscular effort; the immediate suffocative symptoms, at first very grave, gradually passed away, no effusion into the pleural cavity occurred, and complete recovery took place within a few weeks. On physical examination no evidence of phthisis was found. As a confirmatory test, two milligrammes of tuberculin were injected—an amount sufficient to give a marked reaction in a patient suffering from any form of tuberculosis. No result followed. The conclusion was that the case was an instance of benign non-tuberculous pneumothorax. This was justified by the complete recovery of the patient. This method of excluding phthisis as a cause of any case of pneumothorax seems worthy of notice, *e.g.*, If one had a case of pneumothorax which recovered, and in which no physical evidence of phthisis was present, the presence or absence of a tuberculin reaction might determine the advisability or not of sending the patient to a health resort suitable for phthisis.

**Health Resorts for Phthisis and other Diseases.**—The advantages of Grand Canary as a health resort have recently been put forward in an able paper by Melland.[21] The Canary Islands lie quite close—120 miles only—from the African coast opposite the Sahara desert. One important result of this is that the annual rainfall is very slight, at Grand Canary averaging only nine inches. The farther away from the African coast the greater becomes the rainfall; thus, in the most westerly of the Canary Islands it is 25 inches, whilst in Madeira, much more to the west, the rainfall is very much greater. The climate, then, is very dry. Grand Canary is very mountainous, rising in the centre, which is only 18 miles from the coast, to 6,000 ft. The shore climate is an admirable winter climate, but the summer mountain climate at an elevation of from 1,300 to 3,000 ft. is even superior in the treatment of lung disease. The winter climate at Las Palmas, close to the shore, is on the average warmer and drier, and there is much more sunny weather than we have in England in the summer. The summer climate at high altitudes in Grand Canary is fairly hot, but the heat is little felt because the air is so dry, and one is above the clouds. Observations on patients show that Grand Canary climate is very beneficial in both the active and quiescent first stage of consumption, and also in chronic fibroid phthisis. No benefit has been observed in laryngeal phthisis, and in abdominal tuberculosis. Chronic bronchitis, with or without emphysema, is very much improved, as is also chronic rheumatism. The climate is not suitable for cases of pure spasmodic asthma.

Grand Canary is reached from Liverpool in a week; the hotels are excellent with modern English sanitary arrangements, and living is cheap. Grand Canary is as cheap as the Riviera, and not subject to cold winds; it is a good deal less expensive than Egypt, and far less distant than the South African highlands, whilst it has the advantage over the two latter places in that the climate is not so exceedingly dry.

[19] Lancet, June 19, 1897. [20] Medical Week, Feb. 12, 1897. [21] Medical Chronicle.

